# The societal cost of ‘unwanted’ loneliness in Spain

**DOI:** 10.1007/s10198-024-01724-9

**Published:** 2024-09-28

**Authors:** Bruno Casal, Eva Rodríguez-Miguez, Berta Rivera

**Affiliations:** 1 Faculty of Economics and Business, Department of Economy, University of A Coruña, A Coruña, Spain; 2 Faculty of Economics and Business, Department of Economy, University of Vigo, Vigo, Spain

**Keywords:** Unwanted loneliness, Matching, Cost of illness, QALYs, C01, C83, I10

## Abstract

Unwanted loneliness negatively affects people’s health and quality of life, increasing morbidity and the risk of premature death; this situation can generate major social costs. The aim of this study is to estimate the social costs of loneliness in Spain for 2021: both tangible costs –monetary value of health costs and production losses– and intangible costs –*Quality Adjusted Life Years* (QALYs). To estimate costs not derived from mortality, information from a sample of 400 people with unwanted loneliness was compared with that derived from two samples of the general population obtained from the Spanish National Health Surveys. To estimate the costs associated with premature deaths, the population attributable fraction was calculated using the relative risks estimated in previous survival studies. In the baseline scenario, the tangible costs of unwanted loneliness are estimated to be around 14,129 million euros in 2021, representing 1.2% of Spain’s GDP. Approximately 56.8% of the tangible costs correspond to production losses due to reduced working time, and 43.2% are due to healthcare costs related with increased consultation frequency in healthcare services and higher consumption of medicines related to loneliness. In addition, loneliness generates a reduction in quality of life equivalent to 1.04 million QALYs, which corresponds to 2.8% of the total stock of QALYs of the Spanish population over 15 years of age.

**Supplementary Information:**

The online version contains supplementary material available at 10.1007/s10198-024-01724-9.

## Introduction

In the industrialised countries, there is evidence that the quantity and quality of social relations are deteriorating [1, 2]. This situation is due to multiple factors, including reduced intergenerational cohabitation, the difficulty of relating to others (derived from intrinsic or personal factors), greater social mobility, the trend to marry at a later age, a greater proportion of single-person households, an ageing population and an increase in situations of dependency, among others [3, 4]. These situations generate a situation of “unwanted loneliness”, i.e. a negative feeling that is generally defined as having fewer or poorer quality social relationships than one would like to have, which may or may not be accompanied by a situation of social isolation [5–7]. The public health responses implemented by governments in the worst moments of the COVID-19 crisis, mainly based on restrictions on citizen mobility and social distancing, have further aggravated situations of unwanted loneliness and social isolation [8, 9]. Thus, whilst, in 2016, 12% of EU citizens reported feeling lonely more than half the time (11.6% in Spain), in 2020, during the first months of the pandemic, this percentage increased to 25% (18.8% in Spain) [10].

Various longitudinal epidemiological studies and meta-analyses have generated sufficient evidence to confirm that a situation of social isolation or loneliness impacts negatively on health status–increasing morbidity and risk of death– and could explain a higher use of health resources [11–18]. First, loneliness is associated with an increased risk of anxiety and depression [19–23], which may also contribute to death from other causes [24,24–26]. There is also evidence that a situation of loneliness can contribute to greater physical deterioration and disease. There are several mechanisms through which loneliness affects health. A large body of literature has indicated that risk behaviours (such as smoking or sedentary lifestyles) are one of the main mechanisms explaining the relationship between loneliness and physical disease, notably cardiovascular disease [25, 27, 28] and even diseases such as diabetes [29–31]. Loneliness is also associated with other risk factors, such as higher blood pressure, or a worsening of immune function [32], leading to an increased likelihood of premature death. On the other hand, studies have established that having social relationships helps to improve patient care, contributing to increased access and adherence to health services, as well as to a reduction in hospital stays [33–35].

Physical and mental illness associated with loneliness generates a significant economic burden, resulting from increased use of health and care resources [25, 36–41]. It also has a higher social cost related to its impact on the labour market [42] and on the quality of life and well-being of those who experience it [43]. Among the studies quantifying the economic burden of loneliness, one can distinguish those using cost-of-illness approaches [42–47]; and those that analyse the economic and social cost-effectiveness of making an investment to counter it [44, 45, 48–50].

Literature estimating the cost of loneliness is scarce and very heterogeneous. This heterogeneity makes it difficult to compare results of different studies [51]. Studies differ from the perpective they adopt: health system [46], Public Sector [44], or society [45]; their epidemiological approach: prevalence [46] vs. incidence [45]; and the target population –there is a bias towards the elderly population [44–46] vs. a population-based approach [43]. In addition, the use of different scales to measure loneliness and the sources of analysis –combining results from previous studies or surveys representative of the general population– are other important sources of heterogeneity.

However, the main difference lies in the cost categories included. All studies include health care costs, albeit to a greater or lesser extent (e.g. Fulton and Shawn do not include expenditure on medicines). Production losses or indirect costs are only addressed by [45], together with other costs, and by Michaelson et al. [42], exclusively. Finally, we found only one study that estimates intangible costs [43].

This paper deals with estimating the social costs of unwanted loneliness in Spain in a broad sense. On the one hand, the analysis is not restricted to the elderly, but includes the entire population over 15 years of age. On the other hand, different cost categories have been addressed: health costs, production losses and intangible costs. For this purpose, a specific survey was carried out among people suffering from unwanted loneliness, and two control samples of similar people from the general population who are not in this situation was used as a control samples.

## Methods

### Overview of the analysis

The analysis adopted a *prevalence* and *bottom-up* approach. The estimates include both the so- called *tangible costs* –those that can be valued through a market price system– and the *intangible costs*: reduced quality of life due to the physical and emotional suffering experienced by the person facing unwanted loneliness. Tangible costs are health costs directly related to the health problem (costs of primary and specialised care consultation, hospitalisation or treatment), and production losses, associated with decreased participation in the labour market. Tangible costs will be quantified in monetary units and intangible costs in *Quality Adjusted Life Years* (QALYs). Both the estimation of tangible and intangible costs have been separated into two cost items, depending on whether or not these costs are associated with premature mortality. Costs not related to mortality were estimated by comparing a sample of people suffering from unwanted loneliness with a control sample, drawn from a sample of the general Spanish population. *Hazard Ratios* previously estimated in the literature were used to estimate costs related to premature mortality. All cost items have been updated to the year 2021. Some cost items, such as premature mortality, refer to the years prior to 2020 in order to avoid bias that could be caused by the exceptional circumstances of the COVID-19 crisis.

### Samples

The estimation of the social cost not derived from premature mortality was based on different samples, all of which refer to the Spanish population aged 16 and over. To obtain the prevalence of unwanted loneliness, a sample of 4,004 individuals, representative of the Spanish population in terms of age, gender and habitat size, was contacted by telephone in 2022 (sample contact). For this purpose, simple random sampling was applied as follows: (a) 465,000 randomly generated telephone numbers were loaded into the telephone survey system; (b) a random skip of telephone numbers was produced, and (c) random dialling to the previously defined numbers was performed. The selection of individuals experiencing loneliness was carried out by means of a *brief* questionnaire that collected basic information (place of residence, age and sex) about the person contacted, and included a filter question to identify people in a situation of unwanted loneliness. The filter question was as follows: *Regardless of how much or how little contact you have with other people in your daily life*,* how often do you feel lonely? By feeling lonely we mean that you have fewer relationships with other people than you would like*,* or the relationships you do have do not offer you as much support as you would like*. The response options are presented on a 4-level likert scale: “never”; “hardly ever”; “often” and “always”. Interviewees who respond to the levels “often” or “always” are considered to be suffering from unwanted loneliness, in which case they are asked to complete the full questionnaire designed for cost estimation. Once 400 complete interviews of people suffering from unwanted loneliness had been obtained (case sample), the full questionnaire was stopped, but the contact sample –and the *brief* questionnaire– was continued until a representative sample of 4,004 people was reached, thus obtaining the prevalence rates of loneliness by age and sex in the Spanish population. In order to balance the case sample with the age and sex prevalence rates obtained from the contact sample, weighting factors were applied to all the analyses carried out.

The control samples have been extracted from the 2011/2012 and 2017 editions of the *Spanish National Health Survey* (SNHS) [52, 53]. The SNHS-2017 (latest edition available) is the reference survey that collects most of the variables on healthcare resource use, drug consumption and work situations. These variables are necessary for estimating direct health care costs and part of the costs of production losses associated with unwanted loneliness. The 2011/2012 edition of the SNHS is included to estimate the differences in quality of life (intangible costs), because it has been, to date, the only edition of this survey to include the quality of life instrument EQ-5D, which allows us to quantify the loss in terms of QALYs.

In order to obtain the control samples, and with the aim of increasing comparability with the loneliness sample, the following filtering work was carried out. Firstly, we considered it necessary to eliminate from both SNHSs those individuals suffering from unwanted loneliness. Given that the different editions of the SNHS do not have a question on loneliness, we have used the 11 items of the DUKE-UNC-11 social support scale [54–55], which has also been included in our loneliness survey, as a proxy variable. To do this, the sum of the items is obtained (the value of each item ranges from 1 to 5, with a lower value indicating less support) and, using the *Youden Index* [56], the point of greatest discrimination between the loneliness sample and the control samples is determined. The optimal cut-off point (maximum *Youden Index*) is set at 41 points, for both control surveys –the area under the Roc curve (AUC) was 0.86 (95% CI: 0.85–0.88) in the SNHS-2017, and 0.85 (95% CI: 0.84–0.87) in the SNHS-2011/12–, so that all people with a value below 41 are considered to suffer from unwanted loneliness.

Secondly, we have excluded from the SNHS-2011/12 and the SNHS-2017 those individuals who, having been selected to answer the interview, had to be answered by another person because they were hospitalised due to illness, had a disability that prevented their participation, or were unable to answer because of the language. The reason is that people with these characteristics would not have been able to answer our survey either.

### Loneliness sample questionnaire

The information collected in the loneliness questionnaire (400 participants) is divided into four blocks: (1) loneliness; (2) health and well-being; (3) healthcare resource use; and (4) socioeconomic characteristics. The design of the loneliness questionnaire responds to the need to capture information on relevant variables for the subsequent imputation of a monetary value. The application of *matching* techniques to identify differences between loneliness sample and control samples means that the design of this questionnaire is conditioned by the information contained in the control samples.

The loneliness block includes four questions that collect: (1) length of time the individual has been lonely; (2) The *Three-Item Loneliness Scale* (TIL) [57] derived from the *Revised UCLA Loneliness Scale* [58]; (3) reasons for feeling lonely; and (4) times when the individual feels lonely. The health status and well-being block includes questions aimed at collecting information on: (1) chronic disease status; (2) aspects related to disability and limitations in performing activities of daily living; (3) help or support received; (4) self-perceived health status (5-level scale); (5) the EQ-5D-5 L quality of life questionnaire [59–60]; and (6) the DUKE-UNC-11 scale [54–55]. The Spanish version of the last two scales can be found in the SNHS-2011/12 questionnaire.

In the health resources block, questions are included that collect frequency of visits to the family doctor and specialist doctor, as well as frequency of visits to emergency care and hospitalisation services. Questions on the consumption of medicines are also included. Both in the health block and in the healthcare resource use block –in terms of chronic diseases, frequency of visits to services and consumption of medicines–, subjective questions are included to capture how interviewee’s perceive the importance that loneliness has had on them. The degree of influence is recorded on a likert scale with the following five levels: “not at all”, “very little”, “somewhat”, “quite a lot” and “a lot”. The last block of the questionnaire has the following socio-demographic questions: (a) marital status; (b) education level; (c) employment information (employment status, type of working day); (d) household composition; and (e) net household income.

### General methodology for calculating costs not linked to mortality: matching method

*Matching* techniques were used to calculate costs not related to mortality. The main objective of this methodology is to assess the differences in a set of analysis variables (use of health resources, consumption of medicines, quality of life and reduction in work time), between the sample of people suffering from unwanted loneliness and a control sample. The matching method used was *Nearest Neighbour Matching*; the distance algorithm to estimate the propensity score was CBPS (*Covariate Balancing Propensity Score*); and the ratio was 1:30 without replacement. The following covariates were used as control variables to identify similar individuals: age, sex and level of education.

Once the matching is done, regression models were used to estimate the differences between the sample of people who suffer unwanted loneliness and the selected control samples. The most appropriate models would be selected for each of the outcome variables: *logistic regression* for binary responses (consumption of medicines, full-time employment and part-time employment); *Poisson regression* for count responses (number of consultations, number of times hospitalised and number of days hospitalised); and *inflated zero/one regression* (values of the EQ-5D-5 L).

For the cost analysis, the difference between the predicted values of the analysis variables for the case sample and those for the control samples will be taken into account. In general terms, the incremental cost for each of the cost items will be estimated as the product of (a) the estimated difference between the two samples, (b) the ‘rates’ for each cost unit, and (c) the number of individuals who suffer loneliness, estimated from the prevalences obtained in the field survey.

### Valuation of direct health care costs

#### Consumption of medicines

Matching methods were used to estimate the difference between the case and the control sample with respect to the consumption of medicines prescribed for diseases for which there is evidence that a situation of loneliness increases the risk of suffering from them: (1) tranquilisers, relaxants, sleeping pills; (2) antidepressants, stimulants; (3) medicines for diabetes; (4) heart medication; and (5) medicines for blood pressure. The cost of loneliness was estimated by multiplying the consumption differential for those treatments for which a significant difference is found, by the annual cost of the treatment. Information on prescription billing of the National Health System according to therapeutic indication was used. This register contains information to estimate the average number of doses a person consumes in a year for each therapeutic group of medicines. This figure was applied to the difference in drug consumption and the average price of the therapeutic group.

#### Hospitalisation

Hospital expenditure has been estimated by multiplying the difference between both samples in hospital days (obtained by the matching method) by the average cost per day in hospital. The days of hospitalisation were obtained as the product of the number of times the patient was hospitalised in the last year and the days spent in a hospital during the last admission (the SNHS-2017 only has information on the days of the last admission and, therefore, this was the information collected in the case sample). The average cost per day was obtained by dividing the average cost by the average length of stay of the hospitalisation processes in 2019 (to avoid the effects on comorbidity derived from COVID-19), measured in terms of *Diagnosis-Related Group* (DRG). DRG is a reasonable approximation of the cost of treating patients in hospitals. Patients in the same DRG are assumed to be clinically comparable and to use the same hospital resources. All costs associated with a patient’s stay in hospital are included, such as room and board, nursing care, medical supplies, and any procedures or tests performed during the stay. The costs have been updated to the year 2021 using the annual average of the CPI of the *European Classification of Individual Consumption by Purpose* (ECOICOP) healthcare group in each Autonomous Community.

#### Primary, emergency and specialised care consultations

Once estimated the difference between the two samples in the number of primary care, emergency care and specialised consultations, this difference is multiplied by the average cost for each type of consultation, using the most recent reimbursement tariffs. Since in Spain the reimbursement tariffs depend on regional governments, and these are published in different years, they have all been updated to the year 2021, using the regional price index for the health sector in accordance with the ECOICOP classification. The final tariff results from averaging the updated regional tariffs, taking into account the following particularities. For consultations with the family doctor, the cost of the medical consultation without associated tests and in ordinary hours has been considered. The average rate calculated between the rates for first and subsequent consultations is applied. For emergency consultations, we applied the weighted average tariff between the average tariff for all emergencies attended in 2020 (latest available data) in primary care centres (without emergency transport) and the average tariff for emergencies attended in hospital centres (without emergency transport and without subsequent admission). Finally, to calculate the average fees for specialist consultations, the average fee, weighted by frequency, is considered for first consultations (consultations in which diagnosis, treatment and complementary tests are established) and follow-up consultations. The weighting is carried out taking into account the weight that major diagnostic procedures and minor diagnostic procedures have over the total number of procedures registered in the year 2020 in the specialised care of the *Spanish National Health System* (SNHS).

### Valuing production losses not attributable to mortality

Production losses were estimated adopting the *Human Capital* approach, based on the *Human Capital Theory* [61–63] and widely recommended in international economic evaluation guides. Thus, the loss of production is valued on the basis of the estimated gross wage that the individual would no longer receive as a consequence of experiencing this risk factor (unwanted loneliness). In this paper, this loss has been approximated by estimating the difference in the full-time and part-time employment rates. For this purpose, we have estimated the following equation:

1 $$\:\left[{\text{W}}_{\text{F}\text{T}}\times\:\left({\text{E}\text{R}}_{\text{F}\text{T}/\text{s}}-{\text{E}\text{R}}_{\text{F}\text{T}/\text{c}}\right)\right]+\left[{\text{W}}_{\text{P}\text{T}}\times\:\left({\text{E}\text{R}}_{\text{P}\text{T}/\text{s}}-{\text{E}\text{R}}_{\text{P}\text{T}/\text{c}}\right)\right],$$

where *ER*_*TC/s*_*– ER*_*TC/c*_*y ER*_*TP/s*_*– ER*_*TP/c*_ denote the difference in the full-time (FT) and part- time (PT) employment rate, respectively, between the case sample (s) and the control sample (c), estimated using the matching techniques; and *W*_*FT*_ and *W*_*PT*_ denote the median gross annual wage of full-time and part-time jobs, obtained from the *INE Labour Force Survey* for 2021. The output loss associated with loneliness will be estimated by multiplying the result of Eq. (1) by the estimated number of people in loneliness.

### Valuing production losses due to premature deaths

#### Estimating premature deaths attributable to loneliness

To estimate premature deaths attributable to loneliness, the *Population Attributable Fraction* (PAF) –the proportion of deaths that can be attributed to this risk factor– must be calculated. Following the methodology proposed by *World Health Organization* [64], the PAF in group *i* has been estimated using the following equation:

2 $$PAF_i=\frac{P_{i}\text{\:*}\left(RR_{i}-1\right)}{1+P_{i}\text{\:*}\left(RR_{i}-1\right)},$$

where *P* is the prevalence of unwanted loneliness, and *RR* is the relative risk of death associated with loneliness derived from data from survival studies. Once the PAF has been estimated, the number of annual deaths associated with unwanted loneliness by sex and age group is obtained by multiplying the PAF in that group by the number of deaths in the reference population in the same group.

In our case, *PAFs* are estimated from the study developed by Lara et al. [65], which estimates, for a representative sample of the non-institutionalised Spanish population, the effect of intense loneliness on the total causes of death. In addition to being conducted in Spain, this study is of particular interest because of: (1) the quality of the analysis and publication; (2) the need for our study to have estimates for the general population, avoiding studies that calculate *RRs* only for a specific age group; and (3) the use of validated scales for measuring loneliness. In order to adopt the most conservative approach, the *Hazard Ratio* (HR) of the *adjusted* model of Lara et al. is used in our study (following the formula of Shor et al. [66], we found that HR and RR are equivalent in our study,). According to this, a higher level of loneliness is significantly associated with a higher risk of premature mortality (from all causes of death) for those individuals under 60 years of age (*HR* = 1.29; 95% *CI* = 1.02–1.63), but there is no significant difference for individuals over 60 years of age (*HR* = 0.96; 95% *CI* = 0.89–1.04). Given that Lara et al. only analyse the impact of loneliness for those who experience a *TIL* Scale value of 6 or more points (intense loneliness), in order to calculate premature deaths, we have applied the prevalence of *intense* loneliness estimated in our sample. That is, *P*_*i*_ in Eq. [2], expressed in terms of sex and age group, represents the prevalence of individuals within our sample who have attained a TIL Scale value of 6 or more points. Although we consider the study by Lara et al. [65] to be the most appropriate for the context of our study, the *HR* used is in line with that shown in other studies. Thus Holt-Lunstad et al. [67], in a meta-analysis of 70 studies, estimate an *OR* = 1.26 (95% *CI*:1.04–1.53), when analysing the impact of loneliness on mortality. Stokes et al. [12] estimate a *HR* of 1.31 (95% *CI*: 1.19–1.44) when adjusting for sex, race/ethnicity, education and household income.

#### Calculating production losses

The loss of production is valued by estimating the gross wage that the individual would no longer receive from the time of death until the time when he or she should have left the labour market. The general formula for calculating the production losses for individuals of sex *s* who have died at age *a*_*0*_ would be:

3 $$\begin{aligned}{Production\:losses}_{\left[{a}_{0};s\right]}&=\sum_{a={a}_{0}}^{64}{D}_{\left[{a}_{0},s\right]}*{W}_{\left[a,s\right]}*{TE}_{\left[a,s\right]}\\&\quad*{\left(\frac{1+p}{1+r}\right)}^{a-{a}_{0}}*\prod_{t={a}_{0}}^{a}{S}_{t},\end{aligned}$$

where $\:{D}_{\left[{a}_{0},s\right]}$ represents the number of deaths at age *a*_*0*_ and sex *s*, $\:{W}_{\left[a,s\right]\:\:}$ and $\:{TE}_{\left[a,s\right]}\:$ are the average gross wage and employment rate, respectively, of persons of sex *s* at age *a*_*0*_, *St* is the probability of survival at age *t*, *p* is the average annual labour productivity growth and *r* represents the discount rate.

Given that the data is organised according to age range, the equation has been modified to reflect the characteristics of the data set. To do this, several steps are followed. Firstly, the number of deaths attributable to loneliness is estimated by multiplying the estimated *PAFs* by sex and age bracket (5-year intervals from 16 to 65 years) by the deaths occurring in Spain in these brackets during 2019 (to avoid the COVID-19 effect). The data on deaths are obtained from the INE’s *Death Statistics by Cause of Death*. Secondly, we obtain the transition matrix of the deceased individuals by age and sex, over the time horizon, up to retirement age, if they have not died. The transition probabilities are estimated on the basis of the observed survival rate for that interval in the year 2019. The number of individuals estimated for each age and sex group over the time horizon is multiplied by the employment rate and the average gross wage for each group, in 2021. Survival rates are obtained from the INE *Mortality Tables*, the average annual earnings by sex and age from the INE *Wage Structure Survey* [68] and the employment rate from the INE *Labour Force Survey* (EPA) [69]. Third, the estimated future earnings, for each age and sex group, are multiplied by the annual increase in long-term productivity and discounted to the present. A 1% increase has been considered as an estimate of the average annual labour productivity growth in Spain over the last 30 years [70]. A discount rate of 3% is applied, which is usual in economic evaluation studies [71], and is also the discount rate recommended by the Spanish Ministry of Health for economic evaluation studies in health care [72]. A rate of 0% and 5% was also considered for the sensitivity analysis. Finally, the present value of all accumulated earnings for each age and sex interval over the time horizon is aggregated.

### Valuing intangible costs

#### Loss of QALYs not related to mortality

The EQ-5D-5 L instrument has been used to estimate *Health Related Quality of Life* (HRQoL) losses. There are several reasons for choosing this instrument. Internationally, the EQ-5D is the most widely used HRQoL instrument for measuring QALYs. Moreover, for the EQ-5D-5 L, we have estimated rates based on the preferences of the Spanish population [73]. Thirdly, this questionnaire has been incorporated into the SNHS- 2011/12 in Spain: this is the only time this instrument has been incorporated in an official nationwide survey, which allows us to compare the HRQoL results of our sample with those of the general population. Finally, there is empirical evidence that the quality of life weights resulting from this instrument are not taking into account production losses [74–75], a key result to avoid double counting of costs.

To quantify the annual QALY loss associated with unwanted loneliness, we estimate the difference between the weights of the EQ-5D-5 L for the case sample and the control sample (SNHS-2011/12), using the matching technique, and multiplying this difference by the estimated number of individuals who suffer from loneliness.

#### Loss of QALYs due to premature deaths

The procedure for quantifying the discounted QALY losses arising from premature deaths will be very similar to that for production losses. The intangible costs derived from deaths associated with a situation of loneliness will be calculated on the basis of the potential quality- adjusted life years lost, taking into account the survival rates and HRQoL experienced on average by the Spanish population, by age group and sex, obtained from the SNHS-2011/12. The main differences with the formula used to calculate production losses, shown in Eq. 3, are that: (a) the transition matrix does not cover only up to retirement age, but takes into account the entire life time horizon; (b) $\:{W}_{\left[a,s\right]}*{TE}_{\left[a,s\right]}$ is replaced by the mean value of EQ-5D-5 L, $\:{HRQoL}_{\left[a,s\right]}$; and (c) the term *c* is eliminated, assuming zero growth in long-term quality of life.

## Results

### Prevalence of loneliness

Table 1 shows the prevalence of unwanted loneliness in the Spanish population, by age group and gender, derived from the contact sample. We have estimated an aggregate prevalence of 13.4% (95% CI: 12.3–14.4). Applying this prevalence to the Spanish population over 15 years of age as of 1 July 2021 gives an estimate of 5,380,853 (95% CI: 4,939,141-5,782,408) people with unwanted loneliness. In general terms, the prevalence is higher in women than in men and shows a certain U-shape in relation to age –it is more intense in the early years, becomes milder in the intermediate stages and shows a slight upturn in the final stages of life.

**Table 1:** Prevalence of unwanted loneliness by sex and age (%)

	Men (CI 95%)	Women (CI 95%)	Total (CI 95%)
16–24	19.1 (13.9–24.2)	26.1 (20.1–32.0)	21.9 (18.0-25.9)
25–34	18.5 (13.8–23.1)	14.7 (10.4–18.9)	16.5 (13.4–19.7)
35–44	10.5 (7.3–13.7)	16.0 (12.1–19.8)	13.2 (10.7–15.7)
45–54	11.1 (7.9–14.2)	13.1 (9.7–16.5)	12.1 (9.8–14.5)
55–64	10.0 (6.7–13.3)	14.5 (10.7-18-3)	12.4 (9.8–14.9)
65–74	8.0 (4.4–11.6)	7.6 (4.3–10.9)	7.8 (5.4–10.2)
75 and over	10.2 (5.8–14.6)	15.4 (11.1–19.6)	12.2 (9.2–15.2)
**Total**	**12.1 (10.6–13.5)**	**14.8 (13.3–16.3)**	**13.4 (12.3–14.4)**

#### Results of matching methods: differences between case and control samples

Table 2 shows the result of the estimations carried out to assess the costs not related from premature deaths –except for the incidence of diseases, which is only shown for information purposes, as the cost of diseases is taken from the data on attendance and drug consumption. These estimates were obtained by comparing the case sample and control samples –the characteristics of the different samples are shown in the supplementary material to this paper. The *RR* is shown for the variables of healthcare resource frequentations, drug consumption and disease incidence. For the labour market and quality of life variables, the impact (difference between the case and control samples) is shown directly. Compared to the control samples, the case sample shows a significantly higher frequentation of healthcare services, a higher incidence of loneliness-related diseases and a poorer quality of life (measured with the EQ-5D-5 L). A higher consumption of drugs related to loneliness has also been identified, except for drugs for diabetes and hypertension, which do not show significant differences and have therefore not been considered in the analysis. Although the difference in the incidence of diabetes is significant, it does not appear to have had enough of an impact to generate significantly higher treatment costs. Regarding the labour market, the sample of lonely people has a worse labour market participation, given that it has a lower employment rate, characterized by less full-time employment and more part-time employment (Table 2).

**Table 2:** Estimated coefficients: impact of loneliness on the variables of interest

Dependent variable	Comparison	RR (95% CI)	Robust standard error	Coefficient	*p*-value
***Frequency of services***	*[Poisson regressions]*				
Family doctor consultations (last month)	Loneliness survey vs. SNHS-2017	1.910 (1.695–2.125)	0.110	5.898	< 0.001
Specialist consultations (last month)	Loneliness survey vs. SNHS-2017	3.317 (3.095–3.538)	0.113	10.624	< 0.001
Times hospitalised (last 12 months)	Loneliness survey vs. SNHS-2017	1.773 (1.430–2.115)	0.175	3.280	0.001
Average length of stay (in days) last admission (non- admission counts as 0)	Loneliness survey vs. SNHS-2017	1.267 (0.715–1.819)	0.282	0.841	0.400
Emergency consultations (last 12 months)	Loneliness survey vs. SNHS-2017	2.592 (2.283–2.901)	0.158	6.035	< 0.001
***Consumption of medicines (last 2 weeks)***	*[Logistic regressions]*				
Tranquilizers, relaxants, sleeping pills	Loneliness survey vs. SNHS-2017	3.427 (3.302–3.548)	0.135	11.545	< 0.001
Heart medicines	Loneliness survey vs. SNHS-2017	2.000 (1.596–2.389)	0.228	3.220	0.001
Blood pressure medicines	Loneliness survey vs. SNHS-2017	1.096 (0.858–1.311)	0.148	0.768	0.442
Antidepressants, stimulants	Loneliness survey vs. SNHS-2017	5.640 (5.454–5.824)	0.157	12.478	< 0.001
Medicines for diabetes	Loneliness survey vs. SNHS-2017	1.437 (0.945–1.903)	0.270	1.431	0.152
***Diseases in the last 12 months***	*[Logistic regressions]*				
Myocardial infarction	Loneliness survey vs. SNHS-2017	15.106 (14.594–15.614)	0.294	9.442	< 0.001
Angina pectoris, coronary heart disease	Loneliness survey vs. SNHS-2017	11.739 (11.294–12.179)	0.266	9.553	< 0.001
Other heart diseases	Loneliness survey vs. SNHS-2017	2.595 (2.190–2.990)	0.232	4.326	< 0.001
Diabetes	Loneliness survey vs. SNHS-2017	2.200 (1.864–2.523)	0.206	4.186	< 0.001
Depression	Loneliness survey vs. SNHS-2017	7.388 (7.285–7.490)	0.140	17.567	< 0.001
Chronic anxiety	Loneliness survey vs. SNHS-2017	6.161 (6.050–6.269)	0.142	15.815	< 0.001
Other mental illnesses	Loneliness survey vs. SNHS-2017	1.689 (1.334–2.028)	0.208	2.753	0.006
Stroke (embolism, cerebral infarction, cerebral haemorrhage)	Loneliness survey vs. SNHS-2017	14.363 (13.839–14.886)	0.299	9.085	< 0.001
***Labour market***	*[Logistic regressions]*				
Full-time employment rate	Loneliness survey vs. SNHS-2017	-0.071 (0.087–0.055)	0.008	-8.880	< 0.001
Part-time employment rate	Loneliness survey vs. SNHS-2017	0.024 (0.012–0.035)	0.006	4.090	< 0.001
***Quality of Life***	*[Inflated zeroes/ones regression]*				
EQ-5D-5 L (total score)	Loneliness survey vs. SNHS-2011/12	-0.191 (-0.107 – -0.293)		-5.210	< 0.001

*Note* Matching techniques were used to select controls in the SNHS that were similar to the case sample in terms of gender, age and educational attainment

### Estimated costs of unwanted loneliness

#### Direct health costs

Table 3 shows the estimated costs. Regarding direct costs, these are obtained by multiplying: (a) the tariffs obtained as indicated in the methods section; (b) the total overuse/overconsumption by the lonely population compared to the control sample (derived from the matching methods); and (c) the estimated number of people in an unwanted lonely situation. For the estimation of lonely people in the baseline scenario, the average prevalence is applied. A sensitivity analysis is performed in which an optimistic scenario (the estimated prevalence at the lower end of the interval is applied) and a pessimistic scenario (the estimated prevalence at the upper end of the interval is applied) are considered. In the base case, direct health care costs amounted to a total of 6,101 million euros (91.9% of these costs are related to health service use). The consumption of “tranquillizers, relaxants” and “antidepressants and stimulants” accounted for 92.1% of the total cost of medicine consumption.

**Table 3:** Social cost of unwanted loneliness in Spain, 2021

Cost category	Base case^1^	Optimistic case^2^	Pessimistic case^3^
***Tangible Costs***,*** €*** ***(% of GDP)***	***14***,***141***,***088***,***527*** ***(1.17%)***	***12***,***920***,***272***,***036*** ***(1.07%)***	***15***,***307***,***306***,***840*** ***(1.27%)***
***Direct Health Care Costs***,*** €*** ***(% of GDP; % of Total Health Expenditure)***	***6***,***101***,***440***,***763*** ***(0.51%; 4.71%)***	***5***,***600***,***576***,***223*** ***(0.46%; 4.32%)***	***6***,***556***,***772***,***163*** ***(0.54%; 5.06%)***
**Frequency of services**			
Family doctor consultations	1,018,317,735	934,724,488	1,094,311,595
Specialist medical consultations	3,880,573,565	3,562,019,018	4,170,168,607
Hospital stay	110,089,911	101,052,679	118,305,576
Urgent consultation	596,606,770	547,631,587	641,129,663
**Consumption of medicines**			
Tranquilisers, relaxants	158,591,589	145,572,876	170,426,782
Heart medicines	39,199,699	35,981,813	42,125,050
Antidepressants, stimulants	298,061,495	273,593,760	320,304,890
***Production Losses***,*** €*** ***(% of GDP)***	***8***,***027***,***939***,***457*** ***(0.67%)***	***7***,***313***,***248***,***518*** ***(0.61%)***	***8***,***728***,***675***,***817*** ***(0.72%)***
For reduced working time	7,848,411,655	7,204,139,056	8,434,114,017
For premature deaths^4^	179,527,801.73	109,109,462	294,561,800.06
***Intangible Costs***,*** QALYs*** ***(% of QALYs population)***	***1***,***043***,***005.76*** ***(2.84%)***	***951***,***623.52*** ***(2.59%)***	***1***,***133***,***776.33*** ***(3.08%)***
For reduced quality of life	1,027,743	943,376	1,104,440
For premature deaths	15,262.92 17,866	8,247.64 9,242	29,336.27 37,583

^1^ 3% discount rate is applied to premature deaths and average estimate of the prevalence of loneliness

^2^ 5% discount rate is applied to premature deaths and lower estimate (95% CI) of the prevalence of loneliness

^3^ 0% discount rate on premature deaths and upper estimate (95% CI) of the prevalence of loneliness is applied

^4^ A long-term productivity increase of 1% is assumed

#### Valuation of production losses not linked to mortality

Regarding the estimate of production losses due to reduced working time, Table 3 shows that this implies a loss of 7,848 million euros in the baseline scenario (7,204 million and 8,434 million assuming the lower and upper limit of loneliness prevalence, respectively).

#### Valuation of production losses related to premature deaths

A total of 848 premature deaths associated with unwanted loneliness have been estimated in 2019, considering the average prevalence of loneliness (1,024 and 576 if we consider the upper and lower limit of the estimated prevalence of loneliness, respectively). Table 3 shows the estimated production losses resulting from these deaths. In the baseline scenario (resulting from applying a discount rate of 3% and the average prevalence of loneliness) these deaths generate a loss of 180 million euros, ranging from 109 million euros in the optimistic scenario (discount rate of 5% and the lower limit of the prevalence of loneliness) to 295 million euros in the pessimistic scenario (discount rate of 0% and the upper limit of the prevalence of loneliness).

#### Loss of QALYs not linked to mortality

The difference in the EQ-5D instrument score between the sample of lonely people and the control population has been estimated at 0.19 points. Table 3 shows the QALYs lost as a result of multiplying this difference by the estimated population who suffer from loneliness. During the year 2021 approximately 1.03 million QALYs were lost (baseline scenario). If we take the upper (lower) limit of the estimate of the prevalence of loneliness, the estimated loss is 1.10 (0.94) million QALYs. This loss corresponds to 2.8% (2.6% in the optimistic scenario and 3.0% in the pessimistic scenario) of the total QALYs of the Spanish population aged 15 and over, estimated by applying the quality of life weights, by sex and age group, obtained from the ENSE-2011/12 microdata, to the sex and age distribution of the Spanish population in 2021.

#### Loss of QALYs related to premature deaths

The estimate of QALYs lost due to premature deaths associated with loneliness is, in the base case, a loss of 15,263 QALYs (0.04% of total Spanish population QALYs). The pessimistic (optimistic) scenario which assumes a discount rate of 0% (5%) and an upper (lower) bound of the CI of the prevalence of loneliness, gives a value of 29,336 (8,248) QALYs.

## Discussion

In the baseline scenario, the tangible costs of unwanted loneliness have been estimated at approximately 14 thousand million euros, representing 1.2% of the *Spanish Gross Domestic Product* (GDP) in 2021. Approximately 56.8% of the tangible costs correspond to production losses due to reduced working time and 43.2% are due to direct health care costs (resulting from an increase in both health care and medicine consumption). In terms of intangible costs, unwanted loneliness generates a reduction in quality of life equivalent to one million QALYs. This loss represents the 2.8% of the total QALYs of the Spanish population over 15 years of age.

The results of our study are in line with other studies [43–45, 47] which show an association between loneliness and excess costs, both tangible and intangible. The only exception is the study by Shaw et al. [46], which finds a lower cost for the lonely group when controlling for health and lifestyle (but not when controlling only for basic socio- demographic variables). However, if we start from the hypothesis that loneliness influences health, either directly or through mediators (habits, stress, etc.), the introduction of these variables as control variables will block the effect that we want to estimate. This study also confirms the association between loneliness and an increased risk of heart disease and a higher prevalence of mental health, widely documented in other studies [19, 25]. A more comprehensive comparison with existing literature is unreliable given the heterogeneity of published studies, both in target population, perspective of analysis and cost items incorporated in the estimations.

This study is not without limitations. Firstly, the selection of the contact sample and the interviewing of those people who have stated that they suffer from unwanted loneliness was carried out by means of random telephone interviews. This has had an impact on the under-representation of certain profiles, such as the institutionalized population or people with severe material deprivation (at risk of poverty and/or social exclusion). As a result of these limitations, it is to be expected that the prevalence of people in a situation of unwanted loneliness could be higher than the estimated figures, thus biasing downwards the estimate of the estimated costs. However, the recent *Health Survey of the City of Madrid* for the year 2021 confirms our prevalence of loneliness (13.4%), obtaining an average prevalence of 13.8% for the population over 15 years of age [76].

Secondly, the survey used is cross-sectional (there are no official longitudinal surveys in Spain that allow us to analyse this type of relationship) and, therefore, it has limitations when it comes to identifying causal relationships. Although there is literature supporting the causal relationship between unwanted loneliness and the variables of interest [14, 17], this causality, and its direction, cannot be adequately contrasted with our study. For example, although it is confirmed that lonely population has a higher probability of suffering from certain diseases, it could be that, for some pathologies, it is their suffering that generates or aggravates a situation of loneliness in the individual. This may lead to an increase in the estimated costs. In any case, complementary analyses carried out, aimed at analysing respondents’ perception of the importance of loneliness in diseases and the use of healthcare resources, support this causal relationship. This analysis shows that the percentage of participants who consider that loneliness has had a considerable or great influence on each of the variables analysed is as follows: (a) 21% in the frequentation of services (among those who have used them), ranging from 28% in the frecuency of use of specialized consultation to 15% in the use of emergency services; (b) 20% in the consumption of drugs; and (c) 25% in the illnesses analysed, with depression and anxiety standing out with 58% and 51%, respectively. Regarding the impact of loneliness and its effect on labour market participation, a number of recent studies have provided evidence of this causal relationship. Morris [77] found that loneliness predicts work disability and higher unemployment in middle age in European countries. Von Soest, et al. [78], have analysed 4 waves of longitudinal data from 3,116 Norwegians. They conclude that young adults who reported feeling lonely and/or increasing loneliness were at higher risk of labour market exclusion and lower income in midlife. Similarly, a longitudinal Brithis study of 2,232 young adults found that people who were more lonely were more likely to be unemployed, less prepared for the labour market and had lower job optimism [79]. Although the results of these studies support the hypothesis of a causal pathway from loneliness to labour market participation, our findings must be viewed with caution.

Thirdly, it should be noted that certain cost categories were not included in the estimation, as this information was not registered (or was not sufficientlyt detailed) in the general population surveys that we used as a control samples. Thus, for example, with the exception of pharmaceutical co-payments, private outlays made by people experiencing loneliness have not been included (private cost of social services, the cost of travelling to a doctor’s consultation, etc.). The costs associated with other people in the family environment of the lonely person (e.g. care-related costs) and the public cost of social services in the home, or residential care, have not been included either. Finally, production losses due to sick leave have not been taken into account. In any case, this would not detract from the relevance of the costs attributed to unwanted loneliness obtained in this study, since their non-inclusion would bias the estimated costs downwards. Fourthly, the loneliness sample does not include the occupation code of those who are working. This means that we cannot include the average wage by occupational category in the calculation of production losses.

Last but not least, regarding the estimation of intangible costs, these have been quantified in QALYs, using the EQ-5D-5 L instrument for their measurement. However, this instrument may not fully capture all the consequences of loneliness on people’s quality of life. For example, losses in quality of life resulting from feelings of sadness, lack of self-confidence, loss of self-esteem, etc., may not be adequately identified by this instrument. However, the EQ-5D-5 L has been selected because information on this instrument is available for a representative sample of the Spanish population in the SNHS-2011/12, and because it is the most widely used instrument in economic evaluation for measuring quality of life, which facilitates comparability with other studies.

Given the limitations of this study, it has highlighted the significant burden of loneliness on society. The multiple mechanisms through which this impact occurs suggest that there is no single focus of action to prevent or reduce loneliness and its consequences. Interventions aimed at increasing social support networks and opportunities for social interaction, such as online platforms, volunteer visits to isolated people or the creation of community spaces, should be highlighted [80]. Of particular relevance are those interventions whose main aim is to promote intergenerational communication [81]. Other types of interventions or studies aim to raise the visibility of loneliness in society, such as awareness-raising campaigns or those aimed at persuading policy-makers to put this risk factor on the political agenda. Given the variety of initiatives that could be adopted, with very different costs and outcomes, it is necessary to identify the most cost-effective interventions. It should also be noted that interventions or programmes to combat loneliness are not universally effective, but rather work in specific contexts and under specific conditions. It would be beneficial for future research to adopt a realist approach to evaluate which interventions are effective for whom and under what circumstances [82]. Our study can make a twofold contribution to this complex issue. On the one hand, it can help to make policy makers aware of the major impact of loneliness on society and the specific burden of its various costs. On the other hand, it provides a framework for future studies, both in the area of cost of illness studies applied to health risk factors, and in the development of economic evaluations to identify the most cost-effective interventions.

In conclusion, this study represents an initial attempt to assess the influence of a potentially modifiable risk factor, such as loneliness, on an individual’s health and well-being of the Spanish population. The tangible costs of loneliness were estimated at 14,129 million euros, representing the 1.2% of the Spanish GDP in 2021. In addition, the intangible costs were estimated to be around one million of QALYs, representing 2.8% of the total stock of QALYs of the Spanish population aged 15 and above. It is the intention of the authors that this study will provide a basis for further research from a variety of perspectives, which will continue to explore in greater depth the effects of what is already regarded, along with obesity and stress, as one of the most significant public health issues of the 21st century.

## Electronic supplementary material

Below is the link to the electronic supplementary material.

Supplementary Material 1

## Data Availability

Data from the corresponding author upon a reasonable request.
