# Supplementary material for: The societal cost of ‘unwanted’ loneliness in Spain
Source: Eur J Health Econ. 2024 Sep 28;26(4):605–17. doi: 10.1007/s10198-024-01724-9 (PMC12126358; doi:10.1007/s10198-024-01724-9)
Supplement: Supplementary file 1 — Supplementary Material 1 [file 10198_2024_1724_MOESM1_ESM.docx]

**Supplementary material**

**Table 1.** *Descriptive statistics for case and control samples*

| **Variable** | **Loneliness survey (N=400)** | **SNHS-2017 (N=12,000)** | **SNHS-2011/12 (N=12,000)** |
| --- | --- | --- | --- |
| ***Age (%)*** |  |  |  |
| 16-24 | 4.00 | 6.57 | 5.93 |
| 25-34 | 17.00 | 13.53 | 15.00 |
| 35-44 | 20.00 | 22.99 | 20.87 |
| 45-54 | 25.00 | 23.46 | 21.82 |
| 55-64 | 23.00 | 20.98 | 18.63 |
| 65-74 | 7.50 | 8.38 | 11.52 |
| 75 and over | 3.50 | 4.09 | 6.23 |
| ***Gender (%)*** |  |  |  |
| Men | 45.25 | 44.97 | 45.16 |
| Women | 54.75 | 55.03 | 54.84 |
| ***Education (%)*** |  |  |  |
| Does not know how to read or write / Incomplete primary education | 3.00 | 2.89 | 3.00 |
| Complete primary education | 7.50 | 7.61 | 7.50 |
| First stage of secondary education | 17.50 | 18.07 | 17.50 |
| Upper secondary education | 14.00 | 16.39 | 14.00 |
| Intermediate vocational training or equivalent | 10.50 | 10.99 | 12.25 |
| Advanced vocational training or equivalent | 16.00 | 12.71 | 13.45 |
| Universitary studies or equivalent | 31.50 | 31.34 | 32.30 |
| ***EQ-5D (mean; SD)*** | 0.78 (0.22) |  | 0.94 (0.13) |
| ***Use of healthcare services (mean; SD)*** |  |  |  |
| General practitioner or family doctor consultations (last month) | 0.69 (1.24) | 0.3240 (0.68) |  |
| Specialised medical consultations (last month) | 0.7035 (1.33) | 0.20 (0.63) |  |
| Hospitalisations (last 12 month) | 0.19 (0.57) | 0.10 (0.44) |  |
| Number of hospitalisation days (last 12 month) | 0.51 (2.05) | 0.42 (3.59) |  |
| Uses of emergency services (last 12 month) | 1.08 (2.26) | 0.50 (1.20) |  |
| ***Consumption of medication (%)*** |  |  |  |
| Tranquillisers, relaxants, sleeping pills | 36.59 | 9.40 |  |
| Medicines for the heart | 7.77 | 3.63 |  |
| Medicines for high blood preassure | 19.05 | 15.46 |  |
| Anti-depressants, stimulants | 24.06 | 4.15 |  |
| Diabetes medication | 6.02 | 4.90 |  |
| ***Economic activity (%)*** |  |  |  |
| Full-time employed | 52.50 | 61.33 |  |
| Part-time employed | 6.07 | 8.00 |  |
